# Medical Supply Companies

**Published:** 1897-05

**Authors:** 


### MEDICAL SUPPLY COMPANIES.

There have come into existence business organizations into which any one paying the sum of five or six dollars a year will receive medical attendance free of further charge, while the physicians doing the work are to receive ridiculously low fees for their services.

Great stress is laid upon the charitable nature of these concerns, possibly to induce physicians to give their services, while incidentally a petty fee may be collected by the physician for each prescription made.

The object of these companies is not charity, but purely monetary, and one in which the medical profession is to be prostituted to a trades basis, with business corporations fixing the price for professional skill.

No one is debarred from these companies. Rich and poor alike, so long as they pay ten or twenty cents a week, can receive medical attention without further charge so long as physicians can be found for the service.

To be connected with any of these medical companies will seriously affect the professional standing of physicians, and render them ineligible to membership in any of our societies because of the prominent advertising features, since the names of the doctors are printed on cards, and receive wide circulation as members or employés of these medico-business concerns.

We sincerely hope every honorable physician will use his influence, and warn those not acquainted with the attempt made by these companies to utilize the medical profession for financial or trades purposes, thereby degrading our honored calling.

THE BOARD OF CENSORS OF THE HOMEOPATHIC MEDICAL SOCIETY OF THE COUNTY OF PHILADELPHIA.

